# HDAC6—An Emerging Target Against Chronic Myeloid Leukemia?

**DOI:** 10.3390/cancers12020318

**Published:** 2020-01-29

**Authors:** Hélène Losson, Michael Schnekenburger, Mario Dicato, Marc Diederich

**Affiliations:** 1 Laboratoire de Biologie Moléculaire et Cellulaire du Cancer, Hôpital Kirchberg 9, rue Edward Steichen, L-2540 Luxembourg, Luxembourg; helene.losson@lbmcc.lu (H.L.); michael.schnekenburger@lbmcc.lu (M.S.); dicato.mario@chl.lu (M.D.); 2 College of Pharmacy, Seoul National University, 1 Gwanak-ro, Gwanak-gu, Seoul 08826, Korea

**Keywords:** histone deacetylase 6 inhibitor, personalized treatment, heat shock protein 90α, leukemia stem cells, imatinib resistance, targeted therapy

## Abstract

Imatinib became the standard treatment for chronic myeloid leukemia (CML) about 20 years ago, which was a major breakthrough in stabilizing the pathology and improving the quality of life of patients. However, the emergence of resistance to imatinib and other tyrosine kinase inhibitors leads researchers to characterize new therapeutic targets. Several studies have highlighted the role of histone deacetylase 6 (HDAC6) in various pathologies, including cancer. This protein effectively intervenes in cellular activities by its primarily cytoplasmic localization. In this review, we will discuss the molecular characteristics of the HDAC6 protein, as well as its overexpression in CML leukemic stem cells, which make it a promising therapeutic target for the treatment of CML.

## 1. Introduction

Chronic myeloid leukemia (CML) is a well-known hematological malignancy that is characterized in most patients by the translocation t (9; 22) (q34; q11), leading to the formation of the Philadelphia chromosome carrying the chimeric oncogene *breakpoint cluster region-Abelson (BCR-ABL)*. The slow progression of CML can be stopped if the disease is diagnosed and managed early enough with the reference treatment, imatinib, a first-generation tyrosine kinase inhibitor (TKI). Imatinib acts to block the BCR-ABL protein, which has constitutive tyrosine kinase activity, in its inactive form. However, resistances to imatinib and the second and third generation TKIs frequently develop in patients, leading researchers to synthesize molecules targeting other proteins involved in this pathology, such as histone deacetylase 6 (HDAC6). In this review, we will discuss the characteristics and implication of the HDAC6 protein in CML.

## 2. Chronic Myeloid Leukemia

**Figure 1:**
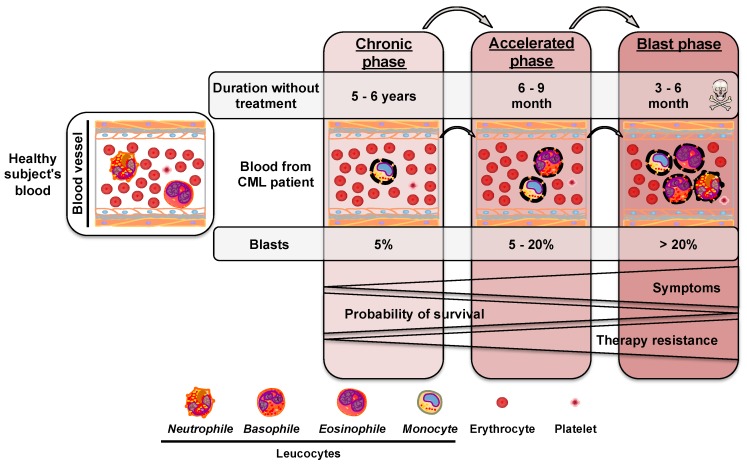
Progression of chronic myeloid leukemia. Chronic myeloid leukemia (CML) is characterized by an excessive proliferation of non-functional leukocytes (dotted lines) or blasts in the bone marrow and then in the blood. The chronic phase remains generally asymptomatic, during which 5% blasts are found in the bloodstream. After 5 to 6 years without treatment, the pathology progresses towards the accelerated phase (5% to 20% blasts) and symptoms including fatigue and loss of appetite and weight begin to appear. Without treatment, the accelerated phase progresses in 6 to 9 months to the blast phase (more than 20% blasts), causing rapid deterioration of the general condition of the patient and death after only a few months without management. Symptoms and resistance to therapy increase during the course of chronic myeloid leukemia, while the probability of survival decreases.

CML is a myeloproliferative disorder affecting myeloid progenitors and is considered as a rare cancer with an incidence of approximately 1 in 100000 cases each year in Europe and the United States [1,2]. There are an estimated 6370 new cases each year in Europe [3] and 8430 new cases have been reported in 2018 in the United States [2]. CML is characterized by an excessive proliferation of leukocytes in the bone marrow, most of which are still immature, and called blasts when they pass into the blood [4]. This pathology comprises three distinct phases: the chronic, accelerated, and blast phases [5] (Figure 1).

### 2.1. Chromosomal Rearrangement during Chronic Myeloid Leukemia

**Figure 2:**
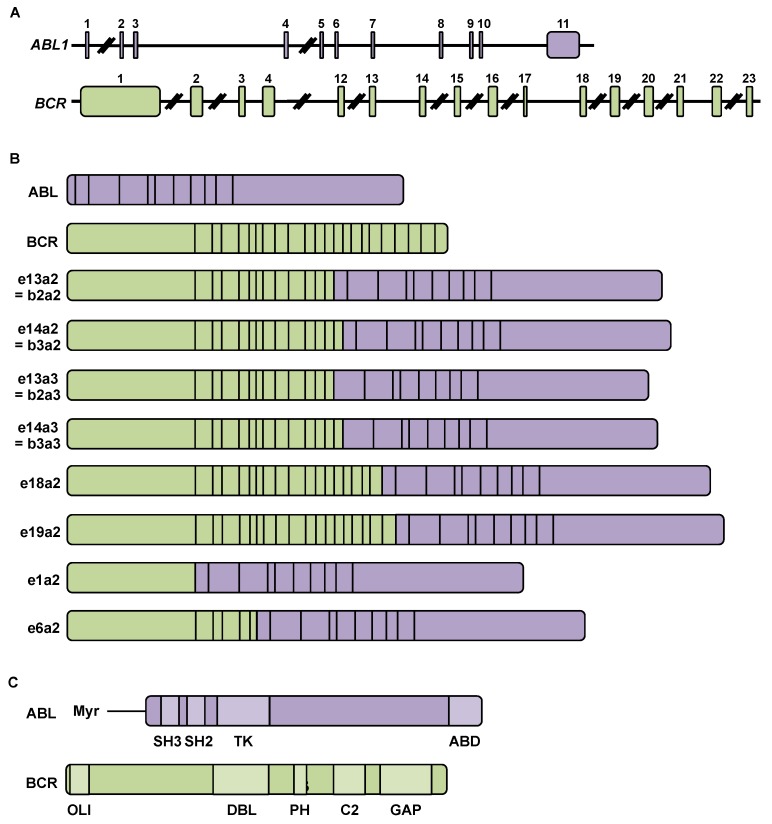
Breakpoints in the *Abelson (ABL)1* and *breakpoint cluster region (BCR)* genes result in the formation of different transcripts encoding the BCR-ABL chimeric protein: (**A**) Structure of the *Abelson* (*ABL*)1 and *breakpoint cluster region* (*BCR*) genes composed of 11 and 23 exons, respectively; (**B**) Diagram of ABL transcripts (1130 amino acids), BCR (1271 amino acids), and BCR-ABL hybrids. The hybrid mRNA eXaY is produced after cleavage occurs after exon X of BCR and before exon Y of ABL; (**C**) ABL protein (145 kDa) has tyrosine kinase activity and predominantly nuclear localization. It comprises a long lipid chain, myristate (Myr), conferring a capacity for self-inhibition, SH2 and SH3 domains of Src homology, allowing interactions with proline-rich regions and with phosphorylated tyrosines, respectively, a tyrosine kinase (TK) domain, and finally an actin-binding domain (ABD) binding domain to DNA and β-actin. ABL is involved in cytoskeletal dynamics (adhesion, polarity, cell migration, and cellular protrusions), proliferation and cell survival, membrane transport (endocytosis, macropinocytosis, phagocytosis, and caveolae formation) by phosphorylating receptors such as epidermal growth factor receptor, platelet-derived growth factor beta receptor and muscle specific tyrosine kinase receptor [8], and finally in autophagy [9,10]. ABL protein is involved in neurodegenerative diseases and neuroinflammation [9,10]. The BCR protein (160 kDa) has serine/threonine kinase activity and is localized in the cytoplasm [11]. BCR comprises an oligomerization domain (OLI). Mutations in this domain cause a decrease in the tyrosine kinase activity of the BCR-ABL protein and prevent the interaction of BCR and BCR-ABL proteins. It also has a domain of homology to the protein DBL [guanosine diphosphate exchange factor (GDP) in guanosine triphosphate (GTP)], a domain of pleckstrin homology (PH), a C2 domain for binding of calcium-dependent phospholipids, and finally a GTPase (GTPase activating protein) domain (GTPase hydrolysis in GDP and phosphate ion) for binding to Rac proteins.

A chromosomal anomaly, the t (9; 22) (q34; q11) translocation between chromosome 9, which carries the *ABL* gene, and chromosome 22, which carries the *BCR* gene, is found in 95% of patients with CML. This translocation leads to the formation of the Philadelphia chromosome carrying the *BCR-ABL* chimeric gene (Figure 2A), and thus to the expression of the corresponding fusion oncoprotein, which has a constitutive tyrosine kinase activity. The Philadelphia or Ph^+^ chromosome was identified for the first time in 1960 by the researchers Hungerford and Nowell in the city of Philadelphia, for which it was named [6,7].

### 2.2. BCR-ABL mRNA and Protein

There are multiple breakpoints on the *BCR* and *ABL* genes that lead to the formation of different transcripts (Figure 2B). These transcripts encode BCR-ABL proteins of different sizes that have been found in patients (Table 1) [4].

The BCR-ABL protein activates many substrates (Table 2) and signaling pathways, including some involved in cell proliferation and survival, through increased activity or expression of a series of anti-apoptotic proteins including the signal transducer and transcriptional activator 5 (STAT5), Akt, phosphoinositide 3-kinase, or B-cell lymphoma-extra-large [20].

## 3. CML Treatments and Associated Resistance Mechanisms

### 3.1. Targeted Therapy with a Tyrosine Kinase Inhibitor

In the 1960s, CML was treated with chemotherapy using busulfan and hydroxyurea. Two different therapeutic strategies emerged in the 1970s: allogeneic stem cell transplantation and the use of interferon-α [5]. A new therapy was approved in 2001 by the Food and Drug Administration (FDA) using the TKI imatinib (Gleevec^®^) [50]. TKIs represent a major advance in the management of CML patients by enabling targeted therapy for the first time.

There are two types of TKIs: type I TKIs target the active and inactive conformation of the BCR-ABL protein and bind to the ATP binding site on the tyrosine kinase domain of the protein, whereas type II inhibitors target the inactive conformation of the protein and bind to a site adjacent to the available ATP binding site when the activation loop is not phosphorylated [51,52].

Imatinib is a type II TKI. Its action therefore prevents the binding of ATP, and thus blocks the protein in its inactive form, preventing its autophosphorylation, and therefore preventing the phosphorylation of its substrates [53]. Imatinib is the first-line treatment for CML with daily oral intake. According to the IRIS study, including 1106 new patients with CML, 76% of patients treated with imatinib also lost the Philadelphia chromosome, also called a complete cytogenetic response. This treatment with a TKI thus allowed stabilization of the pathology in the chronic phase, allowing patients to live normally. However, this treatment has numerous side effects including fatigue, headaches, anemia and myalgia, which are more common, and sometimes even leading to organ toxicity in the liver or heart, which can cause QT interval prolongation associated with a high risk of rhythm disturbances and serious ventricular disease, which are uncommon and occur in less than 1% of patients treated with imatinib [5].

Patients treated with imatinib should be closely monitored to assess their response to treatment. There are three types of responses: the hematological, cytogenetic, and molecular responses. A complete hematologic response corresponds to normalization of blood levels and spleen size. An examination is performed every two weeks until a complete hematologic response is obtained, followed by a control every three months. Then, an examination is performed every 6 months until reaching a complete cytogenetic response, then every 12 to 18 months. Finally, a complete molecular response corresponds to the total disappearance of the BCR-ABL transcript, which is measured by reverse transcription followed by real-time PCR amplification. This test is performed every 3 months according to the recommendations of the European leukemia net [54].

### 3.2. Imatinib Resistances

Although imatinib is the gold standard for CML treatment, 35% to 40% of patients develop resistances to this drug [55]. It is possible to distinguish between primary resistances, defined as a complete lack of hematological response after 3 months, cytogenetic response after 6 months, or a major cytogenetic response after one year of treatment; and secondary resistances, also called acquired resistances, when the previous hematologic or cytological response is lost. Resistances can also be classified according to whether or not they depend on BCR-ABL [56,57]. The emergence of resistances has led to the development of new TKIs (Table 3).

#### 3.2.1. BCR-ABL-Dependent Resistance Mechanisms

**Figure 3:**
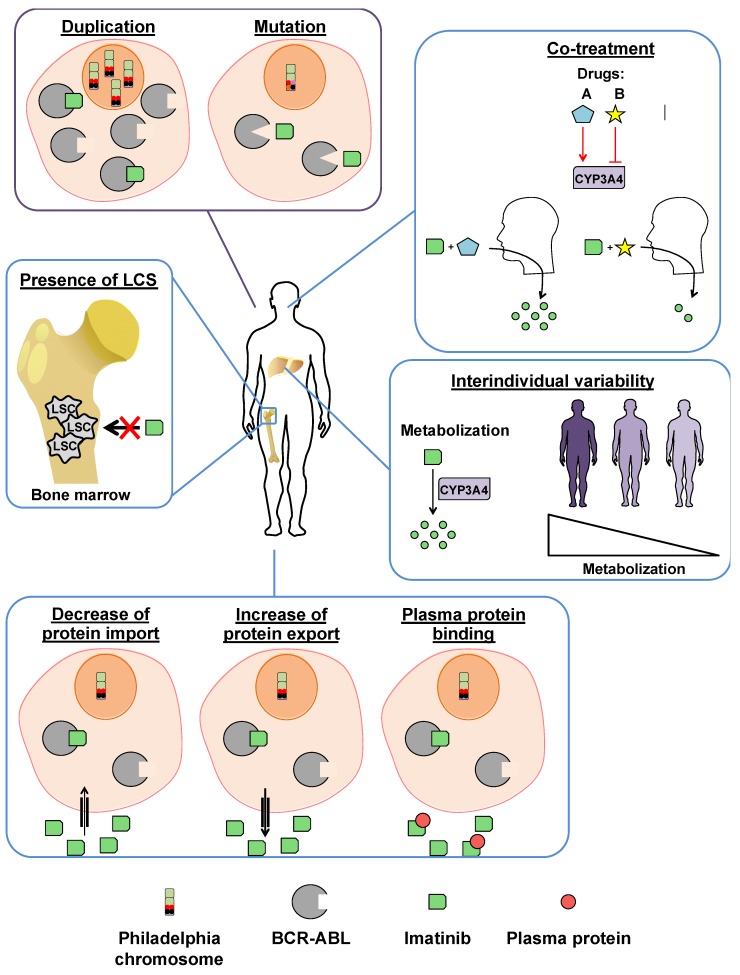
BCR-ABL-dependent and -independent imatinib resistances. BCR-ABL-dependent (purple) and -independent (blue) resistances can be explained by *BCR-ABL* duplication and mutation mechanisms, co-medication, interindividual variety, decreased import proteins, increased export proteins, binding of imatinib to plasma proteins, and the presence of imatinib-insensitive leukemic stem cells (LSCs). CYP3A4: cytochrome 3A4.

BCR-ABL-dependent resistance can be caused by duplication and mutation of the *BCR-ABL* gene (Figure 3). Duplication of the gene has been identified in the cells of imatinib-resistant patients and could be a possible source of drug resistance [63]. Although overexpression of BCR-ABL has been reported in patients with accelerated and blastic phase CML who became resistant to imatinib, several studies have shown that only 3% of imatinib-resistant patients have amplification of *BCR-ABL* gene [64].

Mutations in *BCR-ABL* are more common than duplications and occur in 40% to 90% of imatinib-resistant patients, depending on the sensitivity of the detection method used and the stage of CML [65]. To date, more than a hundred have been discovered [66], which can explain the recently observed decrease in the effectiveness of imatinib treatment [63]. The first mutation described, which is also the most common, represents 14% of all mutations detected [64], and corresponds to the nucleotide substitution of a cytosine by a thymine at position 944 of the *ABL* gene. This mutation results in the substitution of the amino acid 315, initially threonine, with an isoleucine (T315I). This results in the loss of an oxygen molecule that is necessary for the hydrogen bond between imatinib and the tyrosine kinase domain, and also creates steric hindrance, preventing binding and drastically reducing treatment efficacy [67,68,69].

The seven most common mutations are: G250A/E, Y253F/H, and E255D/K/R/V located in the ATP binding P-loop, T315I located at the imatinib binding site, M351T and F359C/L/V/R located in the catalytic loop, and H396P located at the activation loop A [64]. Mutations at the P-loop represent 38% to 46% of all mutations and result in a conformational change that prevents imatinib from binding to BCR-ABL [54]. Mutations occurring at loop A prevent BCR-ABL from attaining its active conformation, thus also preventing binding to imatinib [64]. 

It is interesting to note that the frequency of mutations is higher in patients who have developed secondary resistance, and that the site of mutation varies according to the progression of the pathology. Mutations of amino acids at position 244, 250, and 351 are more frequent in patients in the chronic phase, whereas mutations of amino acids at position 253, 255, and 315 are more frequently encountered in patients in the accelerated or blast phases [64]. 

#### 3.2.2. BCR-ABL-Independent Resistance Mechanisms

BCR-ABL-independent resistances can be explained by interindividual variability, increased export protein, decreased import protein, and also by binding of imatinib to plasma proteins [67].

Interindividual variability may underlie differences in drug metabolism, and thus a different drug response in patients. The metabolization of imatinib to its main circulating metabolite, the N-desmethyl piperazine derivative [55], progresses via cytochrome (CYP) P450, and in particular to the CYP3A4 isoform, which has variable activity. This may explain the observed lower levels of imatinib in some patients despite a similar dose. Cytochrome CYP3A4 can be activated or inhibited by many drugs. The metabolization of imatinib by CYP3A4 may therefore be important during co-medication [65].

A reduced cellular concentration of imatinib can be explained by decreased levels of import proteins or by increased levels of export proteins (Figure 3). The organic cation transporter (OCT)1 is responsible for the influx of imatinib into leukemic cells, and a polymorphism of this transporter is associated with imatinib resistance in K-562 CML cells [64]. Glycoprotein P, also known as multi-drug resistance protein 1, is an export protein that has been associated with failed leukemia treatment by chemotherapy. Recent studies have supported the notion that imatinib is also a substrate for glycoprotein P [70].

Although this hypothesis remains controversial in the literature, the binding of imatinib to plasma proteins such as alpha-1-acid glycoprotein may explain the decrease in free imatinib concentration described in some patients with little or no response to treatment [54,64]. 

Finally, the existence of quiescent leukemic stem cells (LSC), which are unaffected by current drug therapies, would also explain the emergence of resistance to imatinib. LSCs are derived from a group of myeloid progenitors that gain the ability to self-renew, become quiescent, and finally survive in specific microenvironments called hematopoietic niches [4]. These allow interactions between the LSCs and the cells of the microenvironment, which can promote the development of resistance mechanisms. The persistence of LSCs in the presence of TKIs could be the cause of the molecular minimal residual disease that can promote the long-term development of resistance, making them potentially responsible for the clonal evolution and progression of CML [71]. 

### 3.3. Development of Novel Tyrosine Kinase Inhibitors

The emergence of resistances to imatinib has led to the development of new TKIs with an activity against specific mutations in BCR-ABL protein. Dasatinib and nilotinib are second-generation TKIs (Table 3) used in cases of imatinib resistance, allowing a complete, lasting cytogenetic response in approximately 40% of these cases. Despite promising results, these new molecules are nevertheless not effective in patients with the T315I mutation, and lead to the appearance of new mutations generating resistance and subsequently to a decrease in their effectiveness. In addition, the benefit-risk ratio is important to consider as the side effects caused by second-generation TKIs are numerous, and for the most part similar to those generated by imatinib [5].

Ponatinib is a third generation TKI (Table 3) that are effective in second generation TKI refractory patients [72]. Only ponatinib is effective in patients with imatinib resistance associated with the T315I mutation. A complete cytogenetic response was observed in 46% of chronic phase patients receiving ponatinib who had already been treated with other TKIs. Data from a phase II study suggest an increased incidence of arterial thrombotic events in patients receiving ponatinib, thus ending a phase III study. In addition, third generation TKIs do not escape the appearance of resistance phenomena [5,62]. Third generation TKIs also include radotinib, a TKI with structural similarities to imatinib and nilotinib, which was approved in Korea in 2012 for the second-line treatment of CML [73]. This TKI is one of the most effective treatments for chronic phase CML [74] but has side effects including pigment changes such as eruptive melanocytic nevi [75].

## 4. Histone Deacetylase 6

The HDAC6 protein is part of the HDAC family, which are enzymes catalyzing the deacetylation of proteins, which corresponds to the removal of an acetyl group from lysine residues [76]. The 18 HDACs found in mammals are divided into four classes according to their sequence homology. For classes I (HDAC1, 2, 3, and 8), IIa (HDAC4, 5, 7, and 9), IIb (HDAC6 and 10), and IV (HDAC11), the deacetylation of lysine occurs through a transfer of charge, and their essential component is a zinc ion (Zn^2+^) present at the bottom of the catalytic pocket of HDAC enzymes [77]. For class III [sirtuins (SIRT) 1-7] HDACs, the presence of a cofactor, nicotinamide adenine dinucleotide (NAD^+^), is essential for the reaction [78,79].

Class I HDACs are ubiquitously present in many human cell lines and tissues, while class II HDACs exhibit a specific expression profile in certain human tissues such as the heart (HDAC5), the breast (HDAC6), the ovary (HDAC7 and 9), and the kidney (HDAC10) [80,81]. 

### 4.1. Structure

**Figure 4:**
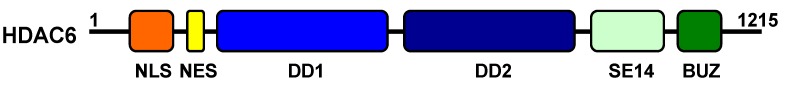
Protein structure of histone deacetylase 6 (HDAC6). The HDAC6 protein consists of 1215 amino acids. It has a nuclear localization sequence (NLS), a nuclear export sequence (NES), two functional active catalytic (SC) sites, a cytoplasmic retention signal of eight consecutive serine-glutamic acid tetradecapeptides (SE14), and a ubiquitin-binding domain (BUZ) at the C-terminal.

Here, we will focus more specifically on HDAC6 belonging to class IIb. This enzyme is the only HDAC to possess two functional active catalytic sites, and has a nuclear localization sequence, a nuclear export sequence, and a repetitive region of eight consecutive serine-glutamic acid tetradecapeptides, a cytoplasmic retention signal, and is mainly present in the cytoplasm [82]. HDAC6 also has a C-terminal ubiquitin-binding domain required when binding to poly-ubiquitinated proteins (Figure 4).

### 4.2. Function 

The HDAC6 protein deacetylates many substrates [83] (Table 4) including α-tubulin, cortactin, and heat shock protein (HSP)90α, and is thus involved in many cell processes, some of which are described below [84].

**Figure 5:**
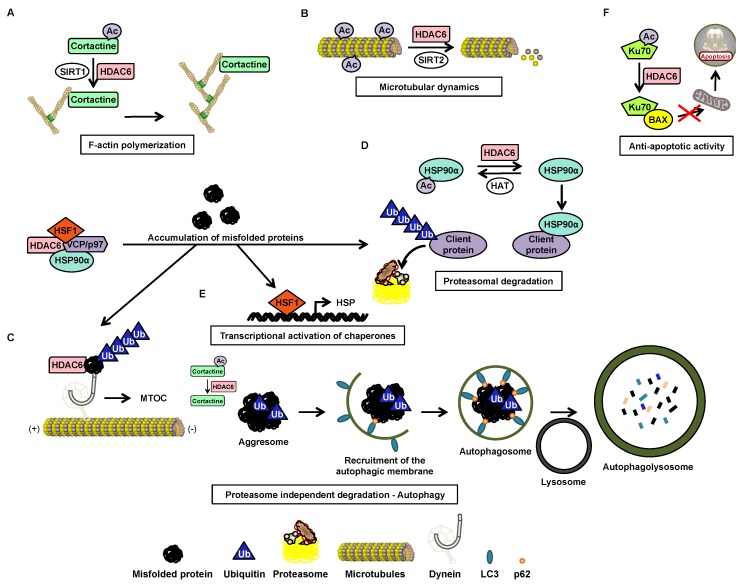
The HDAC6 protein is involved in many cellular processes. HDAC6 is involved in F-actin polymerization (**A**), microtubule dynamics (**B**), anti-apoptotic activity (**C**), proteasome-dependent and -independent degradation (**D**), transcriptional activation of chaperone proteins (**E**), and autophagy (**F**). Ac: acetylated; HAT: histone acetyltransferase; HDAC: histone deacetylase; HSF: heat shock factor; HSP: heat shock protein; MTOC: microtubule organizing center; SIRT: sirtuin; VCP: valosin-containing protein/ATPase.

The HDAC6 protein plays an important role in the dynamism of two components of the cytoskeleton, actin filaments (or F-actin) and microtubules, α- and β-tubulin polymers, which are involved in particular in cell mobility and division. Cortactin, which improves the polymerization of actin filaments, and α-tubulin, a constituent of microtubules, are substrates of HDAC6. The deacetylation of cortactin by HDAC6 and SIRT1 leads to its binding to F-actin, improving its polymerization, and thus contributing to cytoskeletal dynamics (Figure 5A). The deacetylation of α-tubulin by HDAC6 and SIRT2 is associated with microtubule depolymerization, thus contributing to the dynamism of microtubules (Figure 5B), and to proteasome-independent protein degradation. When the proteasome is degraded, the polyubiquitinated misfolded proteins are transported to the microtubule-organizing center and are supported by HDAC6 via its ubiquitin binding domain, leading to the formation of aggresomes through deacetylation of cortactin. The aggresomes thus formed are subsequently removed after autophagosome fusion with lysosomes via autophagy (Figure 5C). A decrease in the acetylation of microtubule-associated protein 1 light chain 3 by HDAC6 was observed during autophagic degradation [68,102]. The HDAC6 protein is also involved in proteasome-dependent protein degradation via its interaction with HSP90α, a chaperone that stabilizes other proteins when deacetylated by HDAC6. In its acetylated form, HSP90α loses its chaperone function, which leads to the degradation of its client proteins by the proteasome (Figure 5D). An accumulation of misfolded proteins causes dissociation of the complex containing HSP90α, heat shock factor 1 (HSF) 1, chaperone valosin-containing protein/ATPase, and HDAC6. In complex in inactive form, during dissociation, the release of HSF1 induces the transcription of many HSPs (Figure 5E), and HDAC6 will allow its binding to misfolded proteins [68].

HDAC6 is also involved in apoptosis by deacetylating the Ku70 protein, which then forms a complex with BAX, a proapoptotic protein, allowing the inhibition of apoptosis (Figure 5F). Similarly, inhibition of the catalytic activity of HDAC6 promotes the dephosphorylation of AKT and ERK, associated with decreased cell proliferation and death of cancer cells [68].

Furthermore, HDAC6 regulates endocytosis and exocytosis vesicles. When the epidermal growth factor receptor (EGFR) receptor is bound to its ligand, it interacts with HDAC6 and inactivates it by phosphorylation, which then leads to the hyperacetylation of microtubules and finally the internalization of the receptor. The inhibition of HDAC6 induces the increase of the acetylation of peroxiredoxins 1 and 2, which are antioxidant enzymes, increasing their activity and causing a reduction in cell resistance to chemotherapy [68]. HDAC6 is involved in the process of autophosphorylation of tau protein, giving it the ability to form aggregates called neurofibrillary tangles that can cause neurotoxicity [103].

### 4.3. Post-Transcriptional Regulation

There is a lack of current data explaining the post-transcriptional regulation of HDAC6 protein. Nevertheless, some microRNAs stimulating cancer cell proliferation and metastasis formation (miR-22, miR-221, miR-433, and miR-548) [84], and stem cell differentiation (miR-26a) [104], are predicted to interact with HDAC6 protein, thus inducing a destabilization or repression of the translation of its mRNA.

### 4.4. Post-Translational Regulation

Post-translational modifications such as phosphorylation and acetylation have a significant impact on HDAC6 functions. Indeed, although EGFR induces an inhibitory phosphorylation of HDAC6, in the majority of cases it is established that the phosphorylation of HDAC6 improves its deacetylase activity, whereas acetylation decreases its enzymatic activity, preventing the deacetylation of α-tubulin. Examples of post-translational modifications of the HDAC6 protein influencing its activity are shown in Table 5.

In addition to these known post-translational modifications, there are proteins interacting directly with the HDAC6 protein and inducing its inhibition by direct interaction (Table 6).

### 4.5. HDAC6 Inhibitors

HDAC6 is overexpressed in many types of cancer (Table 7) and may be implicated in disease progression.

The ability to specifically target HDAC6 would have valuable clinical utility in the treatment of these cancers. However, despite a large number of pan-HDAC inhibitors, very few compounds are capable of selectively inhibiting HDAC6 (Table 8). This type of inhibitor can be divided into 2 groups according to their chemical structure: benzamides and hydroxamates [68]. 

The compounds ACY-241 (Citarinostat) and ACY-1215 (Ricolinostat) are derivatives of hydroxamic acid, which shows a specific inhibitory activity against HDAC6 with IC_50_ values of 2.6 and 5 nM, respectively. They are the only HDAC6 inhibitors in currently clinical trials (Table 9) [130,160]. To date, no HDAC6 inhibitor has yet been approved by the FDA, unlike pan HDAC inhibitors such as romidepsin, SAHA, PXD101, and LBH589 [173].

It is important to note that inactivation of HDAC6 protein in mice does not result in abnormal development or major organ problems [166], suggesting that HDAC6 inhibition would have few side effects, unlike pan-HDAC inhibitors.

## 5. HDAC6 in CML

Several studies have demonstrated the influence of HDAC6 in neurodegenerative, cardiovascular and renal diseases, as well as in inflammation [174] and viral response [84]. The role of the HDAC6 protein in cancer is also now well better understood. Although its oncogenic or tumor suppressor potential is dependent on the type of cancer [68], its involvement in oncogenic cell transformation, tumor development, and cancer immunity regulation makes a strong therapeutic candidate [113]. The overexpression of HDAC6 in many cancer types led researchers to test the effects of HDAC6 inhibitors on these cancers. Despite the observation of a moderate overexpression of the HDAC6 protein in urothelial cancerous tissues, the inhibition of the protein had limited efficacy compared to the use of inhibitors targeting several HDACs [175]. On the other hand, HDAC6 inhibitors have notable anti-cancer properties in prostate cancer [136], breast cancer [176], melanoma [138], and ovarian cancer [102]. These effects could be explained by the implication of HDAC6 in metastasis formation by epithelial-mesenchymal transition induction via its recruitment by TGFβ [177], in cell migration via α-tubulin deacetylation and in angiogenesis via cortactin deacetylation [178]. In contrast to some selective HDAC6 inhibitors, currently approved pan-HDAC inhibitors failed to show any clinical benefits in solid tumors [166]. The reasons of such therapeutic failures, compared to the treatment of leukemia and lymphoma, are not fully understood; however, some hypotheses have been raised. For example, the hypothesis of some researchers is based on the cellular composition of solid tumors, which tend to arise from more differentiated cells with reduced epigenetic reprogramming capacity [179]. In addition, solid tumor complexity including genomic, epigenomic, and phenotypical changes, can be a part of the explanation [180]. Moreover, the lack of response of solid tumors treated with HDAC inhibitors could be due to the pharmacokinetic profile of those drugs, which generally have a short half-life [181]. For such reasons, some researchers are investigating new methods and routes of administration of these inhibitors. Accordingly, Wang et al. have demonstrated that the use of nanoparticles to administrate HDAC inhibitors allowing a slow release of the drug directly in solid tumors could induce a higher therapeutic efficacy than classic administration routes [182]. 

Similar to pan-HDAC inhibitors approved for the treatment of hematological cancers, specific HDAC6 inhibitors showed anti-cancer properties in various cancer types such as multiple myeloma [183], chronic lymphocytic leukemia [114], and acute myeloid leukemia [184]. Here, we will focus on the involvement of HDAC6 in CML.

### 5.1. Nuclear HDAC6 and Its Implication in Leukemia

The cytoplasmic localization of HDAC6 is well described. In leukemia cells, a significant amount of nuclear HDAC6 was revealed, considering the interaction between the nuclear localization sequence (NLS) of HDAC6 and importin-α, which translocates HDAC6 into the nucleus. This region can be heavily acetylated resulting in a reduction of the NLS-importin-α interaction [185]. Consequently, the presence of HDAC6 in the nucleus of leukemia cells could be explained by red levels of acetylation within the NLS region, compared to other cell types. After nuclear translocation, HDAC6 interacts with nuclear proteins, transcriptional repressors, and transcription factors to regulate gene expression. For example, HDAC6 inhibition has been linked to increased expression of the pro-apoptotic protein BIM in acute myeloid leukemia cells [186]. Moreover, in a model exhibiting significant nuclear HDAC6 levels, chemical HDAC6 inhibition reduces its nuclear localization and p53-HDAC6 interactions inducing cell cycle arrest and apoptosis via changes of p53 target gene expression [187]. The specific nuclear localization of HDAC6 in leukemia cells might offer a therapeutic advantage to specifically target those cells.

### 5.2. Degradation of BCR-ABL via Deacetylation of HSP90α by HDAC6 in the Cytoplasm

**Figure 6:**
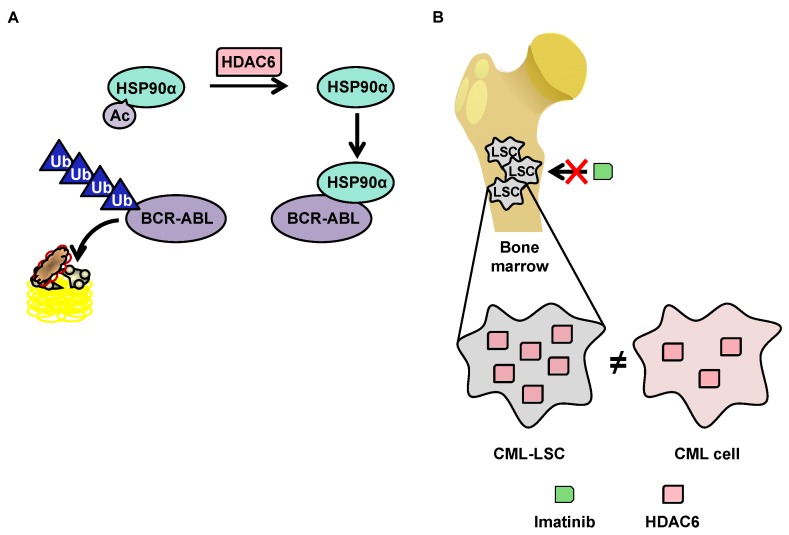
Role of HDAC6 in chronic myeloid leukemia. (**A**) HDAC6 is implicated in proteasome-dependent protein degradation by its interaction with HSP90α, a chaperone protein which, when deacetylated by HDAC6, is involved in the stabilization of BCR-ABL. In its acetylated (Ac) form, HSP90α loses its chaperone function, which leads to ubiquitination (Ub) and subsequent degradation of BCR-ABL by the proteasome. (**B**) Imatinib-insensitive chronic myeloid leukemia (CML) leukemic stem cells (LSCs) overexpress HDAC6 compared to CML cells.

Although little research exists on HDAC6 in the context of CML, this protein has a function that makes it particularly interesting in the context of such pathology. HDAC6 deacetylates HSP90α, which is involved in the stabilization BCR-ABL [186]. In the acetylated form, HSP90α loses its chaperone function, which leads to the degradation of its client proteins by the proteasome (Figure 6A). The importance of the acetylation status of HSP90α in the protein degradation of BCR-ABL makes HDAC6 inhibitors potentially promising molecules for the treatment of CML. Pan-HDAC inhibitors are capable of inducing the inhibition of HDAC6, as well as the downregulation of HDAC6 using si-RNA, which increases the acetylation of HSP90α, and in turn increases the ubiquitination of the BCR-ABL protein, decreasing its expression in K562 cells [188,189].

### 5.3. OverExpression of HDAC6 in CML Stem Cells 

LSCs that are not targeted by TKI and are characterized by a capacity for self-renewal play a crucial role in CML relapse. Although HDAC6 is necessary for the repression of genes involved in the differentiation targeted by the Tip60-p400 complex in embryonic stem cells (ESCs) [190], no study has provided evidence for this in LSCs, more differentiated. In contrast, studies have shown that several proteins in the HDAC family are overexpressed in LCSs of CML. Indeed, SIRT1 is activated by BCR-ABL via STAT5 and its expression is increased in LSCs compared to in CML cells [191]. Finally, overexpression of isoforms of HDAC (HDAC1, HDAC2, HDAC3, HDAC4, and HDAC5) and in particular HDAC6 was more frequently observed in LSCs (CD34^+^ CD38^-^) isolated from patients with CML than in K562 cells [117] (Figure 6B), making it a protein of interest in the search for treatments to prevent relapse in patients with CML.

## 6. Conclusions

Despite the success of imatinib and TKIs, the emergence of resistance and the presence of LSCs that are unaffected by these treatments and are responsible for relapse mean that the development of new treatments for CML remains urgent. HDACs represent potential therapeutic targets for the treatment of CML [188,189,192]. Inhibitors of the HDAC family have promising results in combination with TKIs [193], some of which also inhibit HDAC6 [194,195,196]. The combination of HDAC class I inhibitors [197] or HDAC inhibitors including HDAC6 [198] and TKIs induces elimination of the quiescent LSCs that are not eradicated during treatment with imatinib alone. Although a decrease in HDAC6 expression, an increase in HSP90α acetylation, and a decrease in BCR-ABL expression were observed in imatinib-resistant K562 cells, these cells still showed sensitivity to SAHA, an inhibitor of HDACs including HDAC6 [186]. However, the high toxicity of HDAC inhibitors and their many side effects [183] has necessitated targeting of particular HDAC isoforms.

The targeting of HDAC6 appears promising because it is overexpressed in many types of cancers, including certain leukemia subtypes. A recent study showed that dasatinib inhibits the phosphorylation of BCR-ABL without inducing the death of LSCs, suggesting that LSCs use signaling pathways that are activated by the BCR-ABL protein and are independent of its tyrosine kinase activity. The simple inhibition of the tyrosine kinase activity of the BCR-ABL protein is therefore not sufficient [199]. Since HDAC6 is overexpressed in CML stem cells and that its inhibition can potentially cause BCR-ABL degradation [68], this deacetylase appears as a strong candidate for CML treatment. Accordingly, HDAC6 inhibitors could be further tested in combination with TKIs or other molecules capable of targeting BCR-ABL such as bafetinib, rebastinib, tozasertib, danusertib, HG-7-85-01, GNF-2, and -5 molecules, and 1, 3, 4 thiadiazole derivatives, to potentially reduce resistance to treatment [200]. In the future, further development of selective PROTAC E3 ubiquitin ligase degraders triggering HDAC6 degradation [201] will provide further therapeutic options against various types of cancer, including CML.

## Figures and Tables

**Table 1:** Human BCR-ABL transcripts and proteins. The name and composition of the various human BCR-ABL hybrid transcripts identified in patients are described. The size of the corresponding proteins, their frequency of detection, and the cell lines expressing them are also indicated.

*BCR*-*ABL* mRNA			Size of the Corresponding Protein (kDa)	Examples of CML Cell Lines	References
Hybrid mRNA Name	Composition				
	*BCR* Exons ^$^	*ABL* Exons ^£^			
e13a2 or b2a2	1–13	2–11	210	MEG-01, KBM-7, KYO-1, CML-T1, KCL-22	[12,13,14,15]
e14a2 or b3a2	1–14	2–11	210	K-562, KBM-5, LAMA-84, EM-3, TK-6, EM-2	[12,13,15,16]
e13a3 or b2a3	1–13	3–11	210	NA	[17]
e14a3 or b3a3	1–14	3–11	210	NA	[17]
e18a2	1–18	2–11	225	AR-230	[13,18]
e19a2	1–19	2–11	230	AR-230	[13,17]
e1a2	1	2–11	190	SUP-B15 *, Z-33 *, SD-1 *, TOM-1 *, Z-119 *	[13,14]
e6a2	1–6	2–11	185	NA	[19]

^$^ Of the 23 exons that compose the *BCR* gene. ^£^ Of the 11 exons that compose the *ABL* gene. * Acute lymphocytic leukemia cell lines. ABL: Abelson; BCR: breakpoint cluster region; NA: not applicable.

**Table 2:** BCR-ABL substrates.

Substrates	Phosphorylation site	Function	References
Abi 1 and 2	ND	Proliferation	[21]
BAP-1	Serine and tyrosine residues	Proliferation	[22,23]
Cbl	Tyr-674	Unknown	[22,24,25]
CK2	ND	Cell cycle, apoptosis, transcription, viral infection	[26,27,28]
Crk	Tyr-221	Migration and cellular adhesion	[22,29]
CrkL	Tyr-207	Migration and cellular adhesion	[22,29]
Dok1	Tyr-361	Negative regulation of signaling pathways mediated by tyrosine kinase proteins	[8,22,30]
Fes	ND	Myeloid differentiation	[22,31]
GAP-associated proteins	ND	Ras activation	[22]
GCKR	ND	SAPK activation	[24,32]
Grb2	Tyr-209	Ras activation	[24,33,34]
LASP1	Tyr-171	Interaction with the cytoskeleton, migration and cell survival	[35,36]
Lyn	ND	Cell survival	[37]
Paxillin	ND	Focal adhesion, signaling and cell migration	[22,38]
PLCγ	Tyr-69/Tyr-74	Actin rearrangement and cell migration	[22,39]
PI3-K p85	ND	Proliferation, survival and cellular motility	[22,40]
PKD	Tyr-463	Proliferation, migration and cell survival, angiogenesis, regulation of gene expression	[22,41]
P27Kip1	Tyr-88	Cell proliferation	[42]
p73	ND	Activation of transcription	[22]
Rad9	Tyr-28	DNA damage repair	[22]
Rad51	Tyr-54	DNA damage repair	[22]
Ras-GAP	ND	Apoptosis, proliferation and cell migration	[22,43]
RNA-polymerase II	C-terminal	Transcription	[22]
RAFT1	ND	Cell proliferation, autophagy, cytoskeletal reorganization	[22,44]
Shc	Tyr-427	Migration, angiogenesis	[22,25,45]
SHIP1, SHIP2	Tyr-986 et Tyr-1135 (SHIP2)	Signal transduction, macrophage programming, phagocytosis, migration	[24,25,46]
STAT5	Tyr-694	Signal transduction, transcription activation	[47]
Syp	ND	Unknown	[22]
Talin	ND	Signal transmission between the extracellular matrix and the cytoskeleton	[22,48]
TERT	ND	Genomic integrity	[22,49]
VAV p95	ND	Hematopoietic differentiation	[22]

Abi: Abelson interactor; BAP-1: breakpoint cluster region-associated protein 1; Cbl: Casitas-B-lineage protein; CK: casein kinase; CrkL: Crk like protein; Dok: docking protein; GAP: guanosine triphosphate (GTP)ase-activating proteins; GCKR: germinal-center kinase related protein; Grb2: growth factor receptor-bound protein 2; LASP1: LIM and Src homology (SH)3 protein 1; ND: not determined; PLCγ: phospholipase C-γ; PI3-K: phosphatidylinositol 3-kinase; PKD: protein kinase D; RAFT1: rapamycin and FK506-binding protein (FKBP)-target 1; Ras-GAP: RAS GTPase activating proteins; Shc: Src homology 2 domain containing; SHIP: SH2-containing inositol polyphosphate 5-phosphatase; Syp: SH2-containing phosphotyrosine phosphatase; TERT: telomerase reverse transcriptase; Tyr: tyrosine.

**Table 3:** Tyrosine kinase inhibitors approved by the US Food and Drug Administration for the treatment of chronic myeloid leukemia.

Generation	Drug Name (and Others)	Pharmaceutical Company and Marketing Authorization year by FDA	Targets	Daily Dosage in Adults	References
First	Imatinib ° (Gleevec, STI571, CGP57148B)	Novartis, 2001	BCR-ABL, c-KIT, PDGFR	400 mg single dose	[58,59]
Second	Dasatinib * (Sprycel, BMS-354825)	Bristol-Myers Squibb, 2006	BCR-ABL, Src family, c-KIT, PDGFR	100 mg single dose	[58,59,60]
	Nilotinib ° (Tasigna, AMN107)	Novartis, 2007	BCR-ABL, c-KIT, PDGFR	300 mg in two doses	[58,59,61]
	Bosutinib * (Bosulif, SKI-606)	Pfizer, 2012	BCR-ABL, Src family	500 mg single dose	[58,59,62]
Third	Ponatinib ° (Iclusig, AP24534)	ARIAD Pharmaceuticals, 2012	BCR-ABL, FTL3, Src family, RET	45 mg single dose	[58,59]

° Type II inhibitor binding to the inactive conformation of BCR-ABL. * Type I inhibitor binding to the active and inactive conformation of BCR-ABL. BCR-ABL: breakpoint cluster region-Abelson; FTL3: Fms-like tyrosine kinase 3; PDGFR: platelet-derived growth factor receptor; RET: rearranged during transfection.

**Table 4:** List of substrates specifically deacetylated by HDAC6.

Substrates	Localization of the Substrate	Deacetylated Lysine(s)	Function of the Deacetylated Substrate	Interaction Domains of HDAC6	Reference
14-3-3ζ	Cytoplasm and nucleus	49, 120	Regulation of protein binding Bad and AS160	ND	[85]
β-catenin	Cytoplasm and nucleus	49	Epidermal growth factor-induced nuclear localization and decreased expression of c-Myc	ND	[83]
Cortactin *	Cytoplasm	87, 124, 161, 189, 198, 235, 272, 309, 319	Regulation of cell migration and actin filament binding	DD1 and DD2	[83]
DNAJA1	Cytoplasm	ND	Protein folding	ND	[86]
ERK1	Cytoplasm and nucleus	72	Proliferation, mobility, and cell survival		[87]
Foxp3 *	Nucleus	ND	ND	ND	[88]
HDAC9	Cytoplasm and nucleus	ND	Modulation of cell survival and arrest of cellular movement	DD2	[89]
HDAC11	Nucleus	ND	Transcriptional activation of interleukin 10	ND	[90]
HMGN2	Nucleus	2	Increased transcription of STAT5	ND	[91]
HSC70	Cytoplasm	ND	Protein folding	ND	[86]
HSPA5	Cytoplasm	353	Ubiquitination of HSPA5 mediated by GP78	ND	[92]
HSP90α	Cytoplasm	294	Degradation and elimination of misfolded proteins and regulation of glucocorticoid receptors	DD1, DD2 et BUZ	[83]
K-RAS *	Cytoplasm	104	Cell proliferation	ND	[93]
Ku70	Cytoplasm	539, 542	Suppression of apoptosis	ND	[83]
LC3B-II*	Cytoplasm	ND	Regulation of autophagy	ND	[94]
MSH2	Cytoplasm and nucleus	845, 847, 871, 892	Reduced cellular sensitivity to DNA damaging agents and reduced DNA mismatch repair activities by downregulation of MSH2	DD1	[95]
MYH9	Cytoplasm	ND	Regulation of binding to actin filaments	ND	[86]
PrxI	Cytoplasm and nucleus	197	Antioxidant activity	ND	[96,97]
PrxII	Cytoplasm and nucleus	196	Antioxidant activity	ND	[96,97]
RIG-I	Cytoplasm	858, 909	Recognition of viral RNA	ND	[98]
Sam68	Nucleus	ND	Alternative splicing	ND	[99]
Survivin	Nucleus	129	Anti-apoptotic function	DD2	[83]
Tat	Cytoplasm	28	Suppression of HIV transactivation	DD2 and BUZ	[100]
α-tubulin *	Cytoplasm	40	Formation of immune synapses, viral infection, cell migration and chemotaxis	DD1 or DD2	[83,101]

* Cortactin and LC3B-II are also deacetylated by SIRT1, K-RAS and α-tubulin are also deacetylated by SIRT2, and Foxp3 is also deacetylated by HDAC9 and SIRT1. AS160: Akt substrate of 160 kDa; Bad: Bcl-2 associated agonist of cell death; BUZ: binding-of-ubiquitin zinc; DD: deacetylase domain; DNAJA1: dnaJ homolog subfamily A member 1; ERK1: extracellular signal-regulated kinase 1; Foxp3: forkhead box P3; GP: glycoprotein; HDAC: histone deacetylase; HIV: human immunodeficiency virus; HMGN2: high mobility group nucleosomal binding domain 2; HSC: heat shock cognate; HSP (A): heat shock protein [family A (HSP70) member 5]; LC3B-II: microtubule-associated protein 1 light chain 3; MSH2: MutS protein homolog 2; MYH9: myosin heavy chain 9; ND: non determined; Prx: peroxiredoxin; RIG-I: retinoic acid-inducible gene I protein; Sam: Src-associated substrate in mitosis; STAT: signal transducer and transcriptional activator; Tat: twin-arginine translocation protein.

**Table 5:** Post-translational modifications regulating the activity of HDAC6.

Post-Translational Modification	Enzyme	Target Site	Consequences	Reference
Phosphorylation	GSK3β	Ser-22	Increased deacetylation activity of α-tubulin	[84]
	ERK1	Ser-1035	Regulation of cellular motility	[84]
	GRK2	ND	Increased deacetylation activity of α-tubulin	[105]
	Aurora	ND	Increased deacetylation activity of α-tubulin	[84]
	PKCζ	ND	Increased deacetylation activity of α-tubulin	[84]
	CK2	Ser-458	Improved formation and elimination of aggresomes	[84]
	EGFR	Tyr-570	Inhibition of deacetylation activity	[106]
Acetylation	p300	Lys-16	Inhibition of deacetylation activity	[84]

CK2: casein kinase 2; EGFR: epidermal growth factor receptor; ERK1: extracellular signal-regulated kinase; GRK2: G protein-coupled receptor kinase 2; GSK3: glycogen synthase kinase 3; Lys: lysine; ND: non determined; PKCζ: protein kinase C isoform ζ; Ser: serine; Thr: threonine.

**Table 6:** Proteins that interact directly with the HDAC6 protein.

Protein Inhibiting HDAC6 by Direct Interaction	Protein Function	Protein Region Required for Interaction with HDAC6	HDAC6 Domain Interacting with the Protein	Cellular Impact	References
CYLD	Deubiquitinase	ND	DD1/DD2	Cell proliferation, ciliogenesis	[84]
Dysferlin	Skeletal muscle membrane repair, myogenesis, cell adhesion, intercellular calcium signaling	Domain C2	ND	Myogenesis	[107]
Mdp3	Stabilization factor of microtubules	Amino-terminal region	ND	Cell motility	[108]
Paxillin	Focal adhesion	Region rich in proline	ND	Polarization and cell migration	[84]
p62	Transport of misfolded proteins	Between the ZZ domain and the TRAF6 link area	DD2	Aggresome formation	[109]
RanBPM	Apoptosis, proliferation and cell migration		ND	Aggresome formation	[110]
Tau	Stabilization factor of microtubules	Tubulin binding region	SE14 domain	Aggresome formation	[109,111]
TPPP1	Polymerization and acetylation of microtubules		ND	Regulation of microtubule acetylation and β-catenin expression	[112]

DD: deacetylase domain; Mdp3: microtubule-associated protein (MAP) 7 domain-containing protein 3; ND: non determined; RanBPM: Ran-binding protein microtubule-organizing center; tau: tubulin-associated unit; TPPP1: tubulin polymerization-promoting protein-1.

**Table 7:** Deregulation of HDAC6 expression in different types of cancers.

Cancer Type	Cancers	Expression of HDAC6-Comments	References
Solid tumors	Bladder	Overexpressed	[113]
	Melanoma	Overexpressed	[113]
	Lung	Overexpressed	[113]
	Oral squamous cell carcinoma	Overexpressed-Enhanced expression in advanced stages	[68,114]
	Ovarian carcinoma	Overexpressed-Enhanced expression in advanced stages	[68,114]
	Breast	Overexpressed-Prediction of a good or bad prognosis	[68,115]
	Hepatocytic carcinoma	Overexpressed-Enhanced expression in advanced stages	[68]
		Under-expressed-HDAC6 suggested as a tumor suppressor	[68,116]
Hematological	Chronic lymphocytic leukemia	Overexpressed-Observation on patient samples, cell lines and a transgenic mouse model	[114]
	Acute myeloid leukemia	Overexpressed	[68,114]
	Acute lymphoblastic leukemia	Overexpressed-Enhanced expression in advanced stages	[68]
	Chronic lymphocytic leukemia	Overexpressed-Correlated with longer survival	[68]
	T-cell cutaneous lymphoma	Overexpressed-Correlated with longer survival	[68]
	Chronic myeloid leukemia	Overexpressed-Increased expression in CD34^+^ cells	[117]
	Multiple myeloma	Overexpressed	[118]
	Mantle cell lymphoma	Overexpressed	[118]
	Diffuse large B cell lymphoma	Overexpressed	[118]
	Peripheral T-cell lymphoma	Overexpressed	[118]

CD: cluster of differentiation; HDAC6: histone deacetylase 6.

**Table 8:** List of HDAC6 inhibitors.

Class	HDAC6 Inhibitor	Binding Domain	CI_50_ (nM) of the HDAC6 Activity *in Vitro*	Selectivity Ratio for HDAC6 Compared to (Other HDACs)	Inhibition of HDAC6 *in Cellulo* (µM)^$^	Effect on Cancer Cell Lines or Cancer Type	References
Benzamides	Trithiocarbonate derivative (12ac)	ND	65	19 (HDAC1)	10 (lung cancer)	CI_50_ = 8.2 µM (cervical cancer)	[119]
	NQN-1 (2-benzyl-amino-naphthoquinone)	ND	5540	Values non available (HDAC1, 2, 3, 4, 5, 7, 8, 9, 10, 11)	4 (chronic myeloid leukemia)	CI_50_ = 0.8 µM (leukemia)	[120]
Hydroxamates	Hydroxamic acid containing a phenylalanine (4n)	His215, His216, Tyr386, Phe283, and Tyr255 of DD1 and His610, His611, Tyr782, Phe620, and Phe680 of an HDAC6 homology model	1690	14 (HDAC1)	1 (colorectal carcinoma)	IC_50_: 3 to > 50 µM (various cancer cell lines)	[121]
	Hydroxamic acid containing a pyridylalanine (5a)	Phe566 of DD2 of an HDAC6 homology model	3970	25 (HDAC1)	ND	IC_50_: 104 µM (breast cancer)	[122]
	ACY-738	ND	1.7	55 (HDAC1), 75 (HDAC2), 128 (HDAC3)	2.5 (neural cells)	ND	[123]
	ACY-775	ND	7.5	283 (HDAC1), 343 (HDAC2), 1496 (HDAC3)	2.5 (neural cells)	ND	[123]
	ACY-1083	His573 and His574 of DD2	3	260 (HDAC1)	0.03 (neuroblastoma)	ND	[124,125]
	Bavarostat	Ser568 of DD2	60	>10000 (HDAC1, 2, 3), 188 (HDAC4), 317 (HDAC5), 78 (HDAC7), 142 (HDAC8), 87 (HDAC9), >17 (HDAC10), 167 (HDAC11)	10 (neural progenitor cells derived from induced pluripotent stem cells)	ND	[126]
	BRD9757	ND	30	21 (HDAC1), 60 (HDAC2), 23 (HDAC3), 727 (HDAC4), 611 (HDAC5), 420 (HDAC7), 36 (HDAC8), >1000 (HDAC9)	10 (cervical cancer)	ND	[127]
	Cay10603	His499 of DD2 of an HDAC6 homology model	0.002	ND	<1 to 1 µM (several pancreatic cancer cell lines)	ND	[128,129]
	Citarinostat (ACY-241)	ND	2.6	14 (HDAC1), 17 (HDAC2), 18 (HDAC3 and 4), >7000 (HDAC4, 5,9), 2808 (HDAC7), 53 (HDAC8),	0.3 (ovarian cancer)	CI_50_: 4.6 to 6.1 µM (ovarian and breast cancer)	[130]
	α3β-cyclic tetrapeptide (23)	ND	39	3 (HDAC1), 4 (HDAC3), 6 (HDAC8)	2 (acute lymphoblastic leukemia)	IC_50_: 9 to > 20 µM (various cancer cell lines)	[131]
	Compound containing a phenylisoxazole group as a surface recognition group (7)	His499 of HDAC7	0.002	>100000 (HDAC1), >100000 (HDAC2), 210 (HDAC3), >3000000 (HDAC8), 45350 (HDAC10)	ND	IC_50_: 0.1 to 1 µM (various prostate cancer cell lines)	[128]
	Compound containing a triazolylphenyl group (6b)	ND	1.9	52 (HDAC1), 155 (HDAC2), 7 (HDAC3), 420 (HDAC8), 59 (HDAC10)	ND	IC_50_: <0.5 to 22 µM (several prostate cancer lines)	[132]
	Compound containing a peptoid (2i)	Tyr301 of DD2 of an HDAC6 homology model	1.59	126 (HDAC2), >6000 (HDAC4), 40 (HDAC11)	N	IC_50_: 0.34 to 2.7 µM (various cancer cell lines)	[133]
	3-aminopyrrolidinone derivative (33)	ND	17	4359 (HDAC1), 11 (HDAC8)	0.3 (multiple myeloma)	Good oral bioavailability	[134]
	4-aminomethylaryl acid derivative (1a)	ND	19	305 (HDAC1), 842 (HDAC2), 237 (HDAC3), 790 (HDAC4), 174 (HDAC5), 242 (HDAC7), 36 (HDAC8), 195 (HDAC0)	0.46 (cervical cancer)	ND	[135]
	4-hydroxybenzoic acid derivative (7b)	ND	200	>50000 (HDAC1, 2, 8), >500000 (HDAC3, 10, 11)	50 (prostate cancer)	IC_50_: 41 to 130 (several prostate and breast cancer cell lines)	[136]
	4-hydroxybenzoic acid derivative (13a)	ND	20000	25 (HDAC1), >5000 (HDAC2, 3, 4, 8, 10), >2500 (HDAC11)	50 (prostate cancer)	IC_50_: 19 to 127 (several prostate and breast cancer cell lines)	[136]
	Aminoteraline derivative (32)	Phe620 and Phe680 of an HDAC6 homology model	50	126 (HDAC1), 2 (HDAC8)	2 (neuroblastoma)	IC_50_ = 5.4 µM (neuroblastoma)	[137]
	Benzothiophene derivative (39)	ND	14	ND	Same effect as tubastatin A	Does not target NF-κB and AP-1 at the transcriptional level	[138]
	2,4-imidazolinedione derivative (10c)	ND	4.4	218 (HDAC1), 63 (HDAC2), 53 (HDAC3), > 20000 (HDAC4, 7, 8, 9, 11), 3386 (HDAC5), 37 (HDAC10)	1.6 (acute myeloid leukemia)	IC_50_: 0.2 to 0.8 µM (various cancer cell lines)	[139]
	Mercaptoacetamide derivative (2)	ND	95.3	34 (HDAC1), 77 (HDAC2), 64 (HDAC8), 112 (HDAC10)	ND	At 10 µM protects cortical neurons from oxidative stress inducing death	[140]
	N-Hydroxycarbonylbenylamino quinoline derivative (13)	ND	0.291	32817 (HDAC1), 42955 (HDAC2), 26632 (HDAC3), 15250 (HDAC4), 10694 (HDAC5), 2436 (HDAC7), 4089 (HDAC8), 5258 (HDAC9), 33646 (HDAC10), 1292 (HDAC11)	0.1 (multiple myeloma)	IC_50_: 9.1 to 40.6 µM (multiple myeloma)	[141]
	Isoxazole-3-hydroxamate derivative (SS-208)	His463, Pro464, Phe583, and Leu712 of DD2	12	116 (HDAC1), 1625 (HDAC4), 576 (HDAC5), 695 (HDAC7), 103 (HDAC8), 3183 (HDAC9), 427 (HDAC11)	5 (melanoma)	ND	[142]
	Phenothiazine derivative (7i)	Phe620 and Phe680 of DD2	5	538 (HDAC1)	0.1 (acute myeloid leukemia)	ND	[143]
	Phenylhydroxamate derivative (2)	Phe464 and His614 of DD2	3	27 (HDAC1)	ND	CI_50_: 0.65 to 2.77 (ovarian cancer and squamous cell carcinoma of the tongue)	[133,144]
	Phenylsulfonylfuroxan derivative (5c)	ND	7.4	33 (HDAC1), 51 (HDAC2), 45 (HDAC3), 4 (HDAC4), 46 (HDAC8), 82 (HDAC11)	0.013 (acute myeloid leukemia)	IC_50_: 0.4 to 5.8 µM (various cancer cell lines)	[145]
	Pyridone derivative (11e)	Phe155 and Phe210 of HDAC2	2.46	8 (HDAC1), 52 (HDAC2), 127 (HDAC3), 2329 (HDAC4), 785 (HDAC5), 1512 (HDAC7), 77 (HDAC8), 2268 (HDAC9), 21 (HDAC10), 22 (HDAC11)	ND	IC_50_: 0.14 to 0.38 µM (various cancer cell lines)	[146]
	Pyrimidinedione derivative (6)	ND	12.4	138 (HDAC1), 444 (HDAC2)	ND	Induces arrest of the cell cycle in subG1 phase and death by apoptosis (colon cancer)	[138,147]
	Quinazolin-4-one derivative (3f)	ND	29	65 (HDAC1), 222 (HDAC2), 60 (HDAC18), 141 (HDAC11)	Increases acetylation levels of α-tubulin and histone H3 at 10 μM	ND	[148]
	Sulfone derivative (36)	ND	8	138 (HDAC8), 300 (HDAC11)	0.01 (unspecified)	ND	[138]
	Trichostatine A derivatives (M344, 16b)	ND	88	3 (HDAC1)	ND	ND	[149]
	Tubacin derivative (WT-161)	Phe200, Phe201, Leu270, Arg194 of HDAC7	0.4	129 (HDAC3)	0.3 (multiple myeloma)	IC_50_ = 3.6 µM (multiple myeloma)SangtingTaoCI_50_: 1.5 to 4.7 µM (multiple myeloma cell lines)	[150]
	Tubastatin A derivative (Marbostat-100)	Asp649, His651 et Asp742 of DD2	0.7	1106 (HDAC2), 247 (HDAC8)	0.05 (acute monocytic leukemia)	Non-cytotoxic	[151]
	Indolylsulfonylcinnamic hydroxamate (12)	ND	5.2	60 (HDAC1), 223 (HDAC2)	0.1 (colon cancer)	IC_50_: 0.4 to 2.5 µM (multiple cancer cell lines)	[152]
	MAIP-032	DD2	58	38 (HDAC1)	ND	CI_50_: 3.87 µM (squamous cell carcinoma line of the tongue)	[153]
	MPT0G211	ND	0.291	ND	0.1 (neuroblastoma)	ND	[103]
	N-hydroxy-4-[(N(2-hydroxyethyl)-2-phenylacetamido)methyl)-benzamide)] (HPB)	His573 and His574 of DD2	31	37 (HDAC1)	8 (prostate cancer)	ND	[124,154]
	N-hydroxy-4-(2-[(2-hydroxyethyl)(phenyl)amino]-2-oxoethyl)benzamide (HPOB)	Binding to zinc ion only via its OH group but does not displace the zinc-bound water molecule	56	52 (HDAC1)	16 (prostate cancer, adenocarcinoma, glioblastoma)	Increases the effect on cell viability in combination with etoposide, dexamethasone or SAHA	[155,156]
	N-hydroxy-4-(2-methoxy-5-(methyl(2-methylquinazolin-4-yl)-amino)phenoxy)butanamide (23bb)	Tyr298 and Glu255 of an HDAC6 homology model	17	25 (HDAC1), 200 (HDAC8)	0.051 (cervical cancer)	IC_50_: 14 to 104 nM (various cancer cell lines)	[157]
	Nexturastat A	DD2 of an HDAC6 homology model	5	604 (HDAC1)	0.01 (murine melanoma)	IC_50_ = 14.3 µM (melanoma)	[129,158]
	Oxazole hydroxamate (4g)	Phe620, Phe680, Leu749, and Tyr782 of DD2 of an HDAC6 homology model	59	237 (HDAC1, 8)	10 (cervical cancer)	IC_50_ = 10.2 µM (acute myeloid leukemia)	[159]
	Ricolinostat (ACY-1215)	DD2 of an HDAC6 homology model	4.7	12 (HDAC1), 10 (HDAC2), 11 (HDAC3), 1490 (HDAC4), 1064 (HDAC5), 298 (HDAC7), 21 (HDAC8), >2000 (HDAC9, 11)	0.62 (multiple myeloma)	CI_50_: 2 to 8 µM (multiple myeloma cell lines)	[129,160,161]
	Sahaquine	ND	ND	ND	0.1 (glioblastoma)	CI_50_: 10 µM (glioblastoma)	[162]
	TC24	Ser568, His610, Phe679 and Tyr782 of HDAC6	ND	ND	1 et 10 (gastric cancer)	CI_50_: 10.2 to 17.2 µM (several gastric cancer cell lines)	[163]
	Tetrahydroisoquinoline (5a)	ND	36	1250 (HDAC1), >1000 (HDAC2, 4, 5, 7, 10, 11), 1278 (HDAC3), 58 (HDAC8)	0.21 (cervical cancer)	ND	[135]
	Thiazole	ND	52	ND	ND	ND	[135]
	Tubacin	DD2 of an HDAC6 homology model	4	350 (HDAC1)	5 (prostate cancer)SangtingTao2.5 (acute lymphoblastic leukemia)	IC_50_: 1.2 to 2 µM (acute lymphoblastic leukemia)	[129,164,165]
	Tubastatin A	His610, His611, Phe679, Phe680 and Tyr782 of HDAC6	15	1093 (HDAC1)	2.5 (unspecified)	ND	[163,164]
	Tubathian A	ND	1.9	5790 (HDAC1)	0.1 (ovarian cancer)	ND	[166]
Other	3-hydroxypyridine-2-thione (3-HPT)	Tyr306 of HDAC8	681	5 (HDAC8)	ND	Inactive against two prostate cancer cell lines and one acute T cell leukemia cell line	[167]
	1-hydroxypyridine-2-thione (1HPT)-6-carboxylic acid	DD	150	287 (HDAC1), 4733 (HDAC2), 473 (HDAC4), 233 (HDAC5), 1933 (HDAC7), 22 (HDAC8), 313 (HDAC9)	ND	CI_50_: 18 to 75 µM (leukemia)	[168]
	Adamantylamino derivative (20a)	ND	82	46 (HDAC1), 51 (HDAC4)	ND	ND	[169]
	Mercaptoacetamide derivative (2b)	ND	1.3	3615 (HDAC1)	10 (primary rat cortical culture)	ND	[170]
	Sulfamide derivative (13e)	ND	440	>23 (HDAC1)	1 (bladder cancer)	ND	[171]
Undefined structure	CKD-506	ND	5	>400 (HDAC1, 2, 7, 8)	0.03 (Human PBMCs)	ND	[172]

Arg: arginine; Asp: aspartic acid; CI_50_: concentration inhibiting 50% of cell viability; DD: deacetylase domain; Glu: glutamic acid; HDAC: histone deacetylase; His: histidine; IC_50_: concentration inhibiting 50% of cell growth; Leu: leucine; ND: non determined; PBMC: peripheral blood mononuclear cell; Phe: phenylalanine; Pro: proline; Ser: serine; Tyr: tyrosine.

**Table 9:** HDAC6 Inhibitors in Clinical Trials in Cancer. Clinical studies include four phases. Phase I is performed on healthy volunteers to determine the maximum tolerated dose in humans. Phase II is performed on a limited patient population to determine the optimal dosage. Phase III is performed on several thousand patients and will demonstrate the therapeutic value of the drug and assess its benefit/risk. Phase IV is performed once the drug is marketed and allows to better characterize its adverse effects.

HDAC6 Inhibitor	Clinical Trial Identification	Phase of the Clinical Trial	Pathology
ACY-241	NCT02400242	Ia/Ib	Multiple myeloma
	NCT02935790	Ib	Stage III and IV unresectable melanoma
	NCT02551185	Ib	Advanced solid tumors
	NCT02635061	Ib	Non-resectable non-small cell lung cancer
ACY-1215	NCT02632071	Ib	Unresectable or metastatic breast cancer
	NCT02787369	Ib	Relapsed chronic lymphocytic leukemia
	NCT02091063	Ib/II	Relapsed or refractory lymphoid malignancies
	NCT01997840	Ib/II	Recurrent and refractory multiple myeloma
	NCT01583283	I/II	Multiple myeloma recurrent or recurrent and refractory
	NCT02189343	Ib	Recurrent and refractory multiple myeloma
	NCT01323751	I/II	Multiple myeloma recurrent or recurrent and refractory
	NCT02856568	Ib	Unresectable or metastatic cholangiocarcinoma
	NCT02661815	Ib	Ovarian cancer, primary peritoneal cancer or platinum-resistant fallopian tubes
